# Treatment outcomes and risk factors of death in childhood tuberculous meningitis in Kandahar, Afghanistan: a prospective observational cohort study

**DOI:** 10.1093/trstmh/trac066

**Published:** 2022-07-28

**Authors:** Bilal Ahmad Rahimi, Najeebullah Niazi, Ahmad Farshad Rahimi, Muhammad Ishaque Faizee, Mohmmad Sidiq Khan, Walter R Taylor

**Affiliations:** Department of Paediatrics, Faculty of Medicine, Kandahar University, Kandahar 3809, Afghanistan; Department of Surgery, Faculty of Medicine, Kandahar University, Kandahar 3809, Afghanistan; Kandahar Tuberculosis Centre, Directorate of Public Health, Kandahar 3809, Afghanistan; Department of Histopathology, Faculty of Medicine, Kandahar University, Kandahar 3809, Afghanistan; Head of Paediatric Ward, Mirwais Regional Hospital, Kandahar 3809, Afghanistan; Mahidol Oxford Tropical Medicine Clinical Research unit (MORU), Mahidol University, Bangkok 10400, Thailand; Centre for Tropical Medicine and Global Health, University of Oxford, OX3 7LG, UK

**Keywords:** Afghanistan, children, meningitis, mortality, TB

## Abstract

**Background:**

Tuberculous meningitis (TBM) is the most severe form of TB. We prospectively documented the treatment outcomes and the risk factors for death in children with TBM from Kandahar, Afghanistan.

**Methods:**

This prospective observational cohort study was conducted from February 2017 to January 2020 in hospitalised TBM children. All the patients were prospectively followed up for 12 mo. Data were analysed by using descriptive statistics, χ^2^ and multivariate logistic regression.

**Results:**

A total of 818 TBM hospitalised patients with median age 4.8 (0.8–14.5) y were recruited. Females accounted for 60.9% (498/818). Upon admission 53.9% (n=441) and 15.2% (n=124) had TBM stages II and III, respectively, and 23.2% (n=190) had focal neurological signs. The case fatality rate was 20.2% (160/794) and 30.6% (243/794) survived with neurological sequelae. Independent risk factors for death were being unvaccinated for BCG (adjusted OR [AOR] 3.8, 95% CI 1.8 to 8.1), not receiving dexamethasone (AOR 2.5, 95% CI 1.5 to 4.2), being male (AOR 2.3, 95% CI 1.5 to 3.6), history of recent weight loss (AOR 2.2, 95% CI 1.3 to 3.9) and having stage III TBM (AOR 2.0, 95% CI 1.2 to 3.3).

**Conclusions:**

TBM continues to cause high morbidity and mortality in Afghan children. Strategies to reduce mortality should emphasise early diagnosis and treatment, routine use of dexamethasone and increased BCG vaccination.

## Introduction

TB, an infectious disease caused by the bacillus *Mycobacterium tuberculosis*, ranks globally in the top 10 causes of death and is the leading cause of death from a single infectious disease.^1^ In 2019, approximately 10 million people had TB and 1.4 million died.^1^ Tuberculous meningitis (TBM) is the most severe form of TB and is associated with high mortality rates of approximately 15–30%, despite anti-TB chemotherapy.^2–5^ TBM is also responsible for neurological sequelae and severe disability in 43–79% of survivors^6–8^ and for socioeconomic hardship.^9^ In 2007, a study in India observed neurological sequelae in 78.5% of patients, including cognitive impairment (55%), motor deficit (40%), optic atrophy (37%) and other cranial nerve palsies (23%).^10^ These sequelae were common in patients with baseline focal motor deficits and impaired consciousness.^10^

Delays in the diagnosis and treatment of TBM increases mortality,^11^ with one Peruvian study reporting a 70% increase in mortality with a treatment delay of >3 d after admission.^12^ Diagnosing TBM by the acid fast staining of a cerebrospinal fluid (CSF) has a very low sensitivity of <10%.^13–15^ CSF culture of *M. tuberculosis* has a sensitivity of approximately 50–60% but is slow, requiring up to 8 wk on solid media and, ideally, requires a biosafety level 3 laboratory.^16^ GeneXpert, a WHO-recommended molecular diagnostic method, has a sensitivity similar to culture (50–60%), but with a run time of 2 h.^17–19^ Chest radiography and neuroimaging studies are useful to support a diagnosis of clinically suspected TBM.^20^

Afghanistan is a resource-poor country. Therefore, diagnostic tools for the rapid diagnosis of TB are limited to Ziehl Neelson (ZN) staining of slides and ancillary diagnostic tools such as chest radiograph.^21^ Molecular methods (e.g. GeneXpert) are not readily available, but this limitation is common in many settings. As a result, algorithms that combine clinical features (e.g. duration of illness) with CSF findings and radiology are used to diagnose TBM.^22,23^ Thwaites et al. used five features in adults to predict TBM over bacterial meningitis (i.e. age, length of history, white blood cell count, total CSF white-cell count and CSF neutrophil proportion). This algorithm was 97% sensitive and 91% specific and was validated in a later prospective study.^22,24^

TB remains a major public health challenge in Afghanistan. TB services (diagnosis and treatment) are free of charge and the public health surveillance system is based on confirmed and clinically suspected cases.^25^ Directly Observed Treatment, Short course (or DOTS) is provided by 71% of the 2857 public health facilities. In 2016, there were approximately 65 000 TB cases and approximately 11 000 deaths (crude death rate approximately 17%). In 2017, approximately 392 272 individuals were tested for TB; 47 406 cases were diagnosed, 19 479 (41.1%) bacteriologically confirmed and the rest were diagnosed clinically.^25^ Overall, 56% were females, approximately 20% (9732) were children and about 26% had extra pulmonary TB.^25^ A small, retrospective, hospital-based study of extrapulmonary TB showed that 23/118 (19.5%) had TBM; the most common TBM symptoms were headache (69.6%), fever (60.9%) and vomiting (60.9%). At the time of presentation, 5/23 (21.7%) patients were unconscious, while 6/23 (26.1%) had neck stiffness. Treatment outcomes were not reported.^26^

Afghanistan follows the WHO-recommended treatment for drug-sensitive TBM for adults and children, which consists of a four-drug regimen (isoniazid, rifampicin, pyrazinamide and ethambutol) for 2 mo, followed by 7–10 mo of rifampicin and isoniazid^27,28^ with high dose adjunctive dexamethasone, tapered over 6–8 wk.^27^ Evidence supporting the use of dexamethasone in adults is small but of high quality,^29^ while, for children, the evidence is weaker and is based on smaller randomised trials with supportive findings from observational studies.^30–33^

To our knowledge, there has never been a prospective study of TBM in Afghanistan. We, therefore, conducted a study to describe the features of paediatric TBM, document treatment outcomes and determine risk factors for death.

## Materials and Methods

### Study design, site and participants

**Figure 1.**
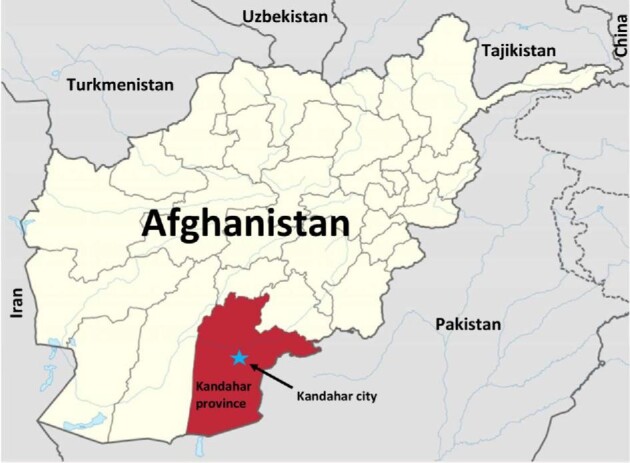
Map of Kandahar, Afghanistan. Source: https://commons.wikimedia.org/wiki/File:Kandahar_in_Afghanistan.svg

This was a prospective observational cohort study that was conducted from February 2017 to January 2020 (3 y) at the Mirwais regional hospital in Kandahar city, the largest tertiary referral hospital in southwest Afghanistan (Figure 1).

Included children had to satisfy the following criteria: (i) a signed consent form by a legal guardian, (ii) age <18 y, (iii) probable or definite TBM as per the scoring system described by Marais et al.,^34^ (iv) admitted to the paediatric ward of Mirwais regional hospital and (v) were residents of Kandahar province. Children were excluded if they had confirmed or CSF findings favouring non-TBM, acute encephalitis or were on anti-TB treatment before admission.

### Clinical care of admitted children

All children underwent a history and physical examination, which included a detailed neurological examination and a lumbar puncture (21- or 23-gauge needles) for standard CSF analysis, including a ZN-stained slide. Blood was taken for routine haematology and biochemistry and repeated as clinically indicated.

### Data collection and analysis

A standardised case record form (CRF) was used to record sociodemographic data, clinical features, laboratory examinations, treatment and outcomes. The CRF was completed by expert paediatricians of the paediatric ward. Histories were taken from the patients’ parents or caretakers. Anonymised data were double entered into Microsoft Excel and cleaned before analysis in SPSS version 22 (Chicago, IL, USA).

Frequencies/percentages and means/medians were used to summarise categorical and continuous variables, respectively. χ^2^ test was used to assess associations of categorical variables and the unpaired t test or non-parametric Mann–Whitney U tests were used to compare continuous data, as appropriate. Statistically significant (p<0.05) variables were assessed for independence by multivariate logistic regression to determine the adjusted OR (AOR) for factors associated with mortality. The cumulative probability of death was determined by Kaplan–Meier survival analysis. p<0.05 was considered statistically significant.

### Treatment and treatment outcomes

All children were treated with anti-TB drugs for 12 mo. For the first 2 mo, intensive therapy comprised isoniazid (INH, 10 mg/kg), rifampicin (15 mg/kg), pyrazinamide (35 mg/kg) and ethambutol (20 mg/kg), followed by 10 mo of maintenance therapy with INH (10 mg/kg) and rifampicin (15 mg/kg). When prescribed, dexamethasone was given for 4 wk: 0.6 mg/kg/day intravenous for 7 d, reducing to 0.3 mg/kg/day for 21 d. Treatment outcomes were assessed at 1 wk, 1 mo, 6 mo and 12 mo using the Paediatric Cerebral Performance Category (PCPC) scale.^35^

## Definitions

**Confirmed TBM:** clinical criteria (symptoms and signs of meningitis, including ≥1 of the following: headache, irritability, vomiting, fever, neck stiffness, convulsions, focal neurological deficits, altered consciousness or lethargy) plus ≥1 of the following: acid-fast bacilli (AFB) seen in the CSF, *M. tuberculosis* cultured from the CSF or a CSF-positive commercial nucleic acid amplification test.^34^

**Probable TBM:** for this, we also used the criteria from Marais et al.: clinical criteria plus a total diagnostic score of either ≥10 points (cerebral imaging not available) or ≥12 points (cerebral imaging available) and exclusion of alternative diagnoses. At least two points should come from either CSF findings or cerebral imaging criteria.^34^

**Staging/grading of TBM severity:** we used the Medical Research Council (MRC) TBM severity grades: (i) Grade I, alert and orientated without focal neurological deficit; (ii) Grade II, Glasgow coma score (GCS) 11–14 with or without focal neurological deficit or GCS 15 with a focal neurological deficit; and (iii) Grade III, GCS≤10 with or without a focal neurological deficit.^36^

**Tuberculin skin test:** using WHO guidelines, the tuberculin skin test (TST) was regarded as positive when there was >5 mm of induration in high-risk children (including HIV-infected and severely malnourished children) or >10 mm of induration in all other children, irrespective of previous BCG vaccination.^37^

**Socioeconomic status: **low socioeconomic status, monthly income <2500 Afghanis (<US$30); middle socioeconomic status, monthly income 2500–20 000 Afghanis (US$30–250); and high socioeconomic status, monthly income >20 000 Afghanis (>US$250).

**Malnutrition^38^:** mild acute malnutrition, weight-for-length/height <–1 SD of the median; moderate acute malnutrition, weight-for-length/height ≥–3 to <–2 SD of the median; and severe acute malnutrition, weight-for-length/height <−3 SD of the median.

**PCPC scale:** according to this scale, 1 was considered to denote normal, 2 mild disability, 3 moderate disability, 4 severe disability, 5 coma or vegetative state and 6 brain death/death.^35^

**Normal laboratory cut-off values**

**CSF^39^:** colour=colourless, clear, like water.

**Glucose:** 45–80 mg/dL.

**Proteins:** age ≤6 d=70 mg/dL; age up to 4 y=24 mg/dL; and age >4 y=20–40 mg/dL.

**Leukocyte count:** age <1 y=0–30/mm^3^; age 1–4 y=0–20/mm^3^; and age ≥5 y=0–10/mm^3^.

**Full blood count^40^**

**Haemoglobin (g/dL):** age <1 mo=15.0–24.0; 1–23 mo=10.5–14.0; 2–9 y= 11.5–14.5; 10–17 y (males)=12.5–16.1; and 10–17 y (females)=12.0–15.0.

**Total leukocyte count (N/mm^3^):** age <1 mo=9100–34 000; 1–23 mo=6000–14 000; 2–9 y=4000–12 000; and 10–17 y=4000–10 500.

**Neutrophils:** 54–62%.

**Lymphocytes:** 25–33%.

**Platelets (Nx1000/mm^3^):** 150–400.

## Results

**Figure 2.**
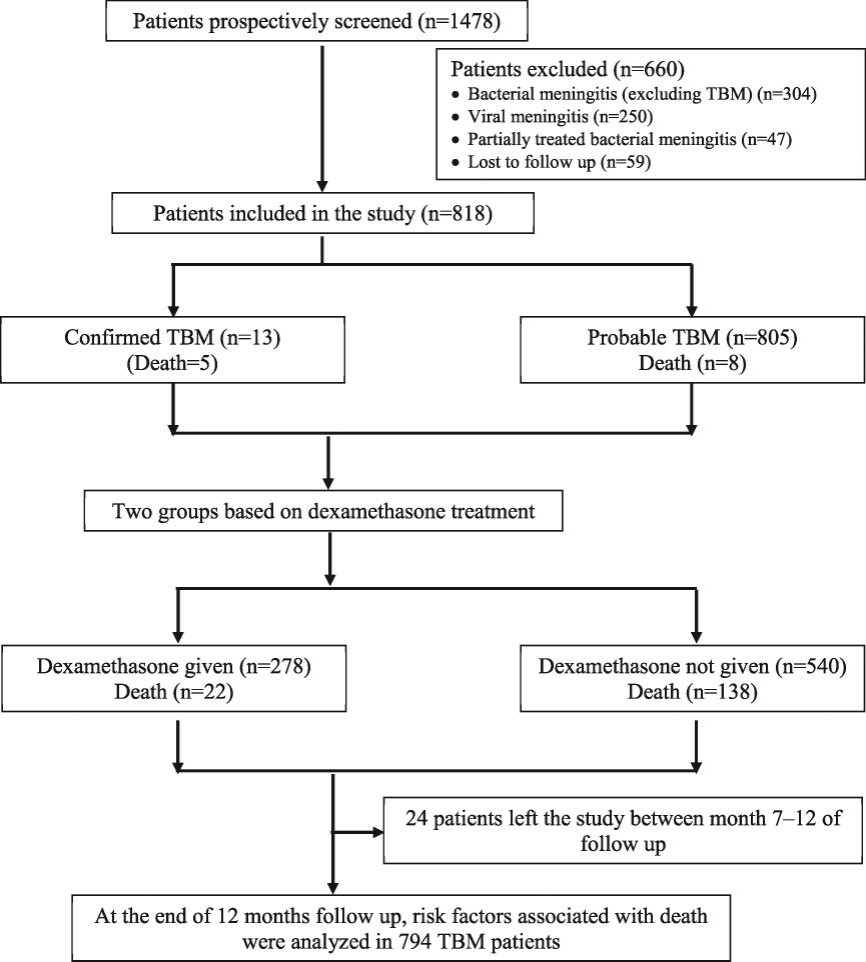
Flowchart of the TBM study.

In total, 818 children met the inclusion criteria and were selected for the final analysis (Figure 2). As each patient was followed up prospectively for 12 mo during anti-TB treatment, this study lasted 3 y. The median admission age was 4.8 y and the majority of the patients (60.9%; 498/818) were female (Table 1). Patients had been ill for a median (range) of 26 (8–45) d prior to hospitalisation.

**Table 1.** Baseline sociodemographic and other features of TBM patients

Variable	All children (N=818)	Dexamethasone (N=278)	No dexamethasone (N=540)	p
Age, y, median (range)	4.8 (0.8–14.5)	4.6 (0.8–13.7)	4.9 (1.1–14.5)	0.807
Age <2 y, N (%)	209 (25.5)	65 (23.4)	144 (26.7)	0.346
Age 2–<5 y, N (%)	325 (39.7)	105 (37.8)	220 (40.7)	
Age 5–<12 y, N (%)	236 (28.9)	89 (32.0)	147 (27.2)	
Age ≥12 y, N (%)	48 (5.9)	19 (6.8)	29 (5.4)	
Female, N (%)	498 (60.9)	176 (63.3)	322 (59.6)	0.532
Nutritional status, N (%)				
Normal	256 (31.3)	87 (31.3)	169 (31.3)	0.574
Mild malnutrition	154 (18.8)	56 (20.1)	98 (18.1)	
Moderate malnutrition	331 (40.5)	105 (37.8)	226 (41.9)	
Severe malnutrition	77 (9.4)	30 (10.8)	47 (8.7)	
Concurrent infections^a^, N (%)	181 (22.1)	54 (19.4)	127 (23.5)	0.182
BCG unvaccinated^b^, N (%)	588 (71.9)	163 (58.6)	425 (78.7)	<0.001
Known TB contact, N (%)	230/797 (28.9)	92 (33.1)	138 (25.6)	0.026
Time to diagnosis, days, median (range)	27 (10–68)	24 (10–51)	30 (14–68)	0.279
Number of people living in the same house (n=809), mean (SD)	12 (6)	11 (6)	13 (6)	0.758
Number of people living in the same room (n=809), mean (SD)	5 (2)	4 (2)	6 (2)	0.863
Number of siblings (n=794), mean (SD)	6 (3)	6 (3)	6 (3)	0.947
Socioeconomic status, N (%)				<0.001
Low	658 (80.4)	196 (70.5)	462 (85.6)	
Middle	129 (15.8)	66 (23.7)	63 (11.7)	
High	31 (3.8)	16 (5.8)	15 (2.8)	

a Concurrent infections were pneumonia (n=65), tonsillitis/pharyngitis (n=36), helminthiasis (n=31), giardiasis (n=28), amebiasis (n=14) and other infections (n=7).

b BCG vaccination was based on history and documentation of vaccine date.

Table 1 shows the baseline sociodemographic and other main features of TBM patients. Most of the patients did not have a known TB contact at home (567/797; 71.1%), had not received BCG vaccination at birth (588/818; 71.9%) and were from families of a low socioeconomic status (658/818; 80.4%). None of the children with a known TB contact had received postexposure prophylaxis.

Table 2 reveals the symptoms and signs of TBM patients. Upon admission, 54.6% of patients (447/818) were in grade II of TBM. Among the patients with cranial nerve palsy, the majority (150/267; 56.2%) had VI nerve palsies.

**Table 2.** Symptoms and signs in patients with TBM

Variable	All children (N=818)	Dexamethasone (N=278)	No dexamethasone (N=540)	p
Symptom duration, d, median (range)	26 (9–51)	24 (9–43)	27 (13–48)	0.864
Fever, N (%)	785 (96.0)	266 (95.7)	519 (96.1)	0.786
Cough >2 wk, N (%)	289 (35.3)	98 (35.3)	191 (68.7)	0.973
Headache (N=534), N (%)	455 (82.1)	148 (53.2)	307 (56.9)	0.815
Recent weight loss, N (%)	128 (15.6)	32 (11.5)	96 (17.8)	0.019
Vomiting, N (%)	201 (24.6)	64 (23.0)	137 (25.4)	0.460
TBM grade upon admission, N (%)IIIIII	253 (30.9)447 (54.6)118 (14.4)	83 (29.9)118 (42.4)77 (27.7)	170 (31.5)329 (60.9)41 (7.6)	<0.001
Impaired consciousness^a^, N (%)	168 (20.5)	64 (23.0)	104 (19.3)	0.207
Focal neurological signs, N (%)	190 (23.2)	40 (14.4)	150 (27.8)	<0.001
Cranial nerve palsy (N=695), N (%)	267 (38.4)	86 (30.9)	181 (33.5)	0.562
Cranial nerve III palsy	41 (15.4)	20 (23.3)	50 (27.6)	0.429
Cranial nerve VI palsy	150 (56.2)	45 (52.3)	92 (50.8)	0.836
Cranial nerve VII palsy	76 (28.5)	21 (24.4)	39 (21.5)	0.438
Neck stiffness (N=658), N (%)	446 (67.8)	148 (53.2)	298 (55.2)	0.304
Seizures^b^, N (%)	285 (34.8)	93 (33.5)	192 (35.6)	0.550
Clonus (N=695), N (%)	194 (27.9)	64 (23.0)	130 (24.1)	0.497
Hypertonia (N=717), N (%)	342 (47.7)	124 (44.6)	218 (40.4)	0.370
Raised ICP^c^ (N=684), N (%)	305 (44.6)	110 (39.6)	195 (63.1)	0.359
Papilledema (N=699), N (%)	299 (44.7)	103 (37.1)	196 (36.3)	0.582
Bulging fontanelle^d^ (N=207), N (%)	43 (20.8)	15 (5.4)	28 (5.2)	0.868
Sunsetting sign (N=207), N (%)	6 (2.9)	2 (0.7)	4 (0.7)	0.980

a Impaired consciousness was defined as a Glasgow Coma Scale score of ≤12.

b Seizures observed upon admission and by history ≤48 h of hospitalisation.

c Diagnosis of raised intracranial pressure (ICP) was based on clinical signs and symptoms: papilledema, bulging fontanelle and sunsetting sign.

d Bulging fontanelle was observed in patients aged <18 mo.

Table 3 shows the laboratory examination upon admission, radiological findings and treatment of TBM patients during hospitalisation. In the CSF analysis, only 165/818 (20.2%) of the patients had turbid CSF.

**Table 3.** Laboratory examination upon admission, radiological findings and treatment of TBM patients during hospitalisation

Variable	Total (N=818)	Dexamethasone (N=278)	No dexamethasone (N=540)	p
CSF findings				
Turbid, N (%)	165 (20.2)	79 (28.4)	86 (15.9)	0.921
White blood cells, N/mm^3^, median (range)	154 (9–869)	163 (51–859)	146 (9–787)	0.731
Lymphocytes, %, median (range)	69 (21–99)	73 (28–99)	64 (21–97)	0.873
White blood cells, 10–500/mm^3^, N (%)	711 (86.9)	229 (82.4)	477 (88.3)	0.582
White blood cells >100/mm^3^, N (%)	471 (57.6)	167 (60.1)	304 (64.5)	0.301
Lymphocyte predominant (>50%), N (%)	680 (83.1)	219 (78.8)	461 (67.8)	0.017
Protein, mg/dL, median (range)	99 (22–580)	105 (31–580)	94 (22–498)	0.694
Protein>100 mg/dL, N (%)	394 (48.2)	132 (47.5)	262 (66.5)	0.779
Glucose, mg/dL, median (range)	39 (8–59)	42 (11–59)	35 (8–57)	0.792
CSF glucose<40 mg/dL, N (%)	339 (41.4)	118 (42.4)	221 (65.2)	0.676
CSF: blood glucose ratio <50%, N (%)	322 (39.4)	111 (39.9)	211 (65.5)	0.813
Full blood count				
Haemoglobin, g/dL, median (range)	10.2 (5.4–13.9)	10.1 (5.4–12.8)	10.8 (6.5–13.9)	0.941
Total leukocyte count, N/mm^3^, median (range)Neutrophils, %, median (range)Neutrophilia (>70%)Lymphocytes, %, median (range)Lymphocytosis (>40%)	11 987 (6350–17 290)64 (15–74)314 (38.6)31 (19–85)68 (8.3)	11 021 (6350–16 498)60 (15–69)98 (35.3)29 (19–85)22 (7.9)	12 895 (7160–17 290)69 (18–74)216 (40.0)33 (22–82)46 (8.5)	0.8730.7510.8520.8270.874
Platelets, Nx1000/mm^3^, median (range)	248 (148–379)	266 (159–379)	228 (148–351)	0.693
TB tests				
Positive TST (N=658), N (%)	128 (19.5)	38/224 (17.0)	90/434 (20.7)	0.389
TST zero reaction, N (%) TST 1–5 mm, N (%) TST 6–10 mm, N (%) TST 11–15 mm, N (%) TST >15 mm, N (%)	476 (72.3)59 (9.0)25 (3.8)68 (10.3)30 (4.6)	171 (76.3)16 (7.1)7 (3.1)21 (9.4)9 (4.1)	305 (70.3)43 (9.9)18 (4.2)47 (10.8)21 (4.8)	
Positive sputum AFB smear (N=207), N (%)	43 (20.8)	15 (22.1)	28 (17.5)	0.868
Chest radiograph (N=672)				
Miliary TB, N (%)	34 (5.1)	14/229 (6.1)	20/443 (4.5)	0.738
Hilar lymphadenopathy, N (%)	196 (29.1)	59/229 (25.8)	137/443 (30.9)	
Pneumonia, N (%)	7 (1.1)	2/229 (0.9)	5/443 (1.1)	
Normal chest radiograph, N (%)	435 (64.7)	139/229 (60.7)	296/443 (66.8)	
Treatment outcomes (N=794)				
Died, N (%)	160 (20.2)	22 (8.3)	138 (26.1)	<0.001
Survived with neurological sequelae, N (%)Survived without neurological sequelae, N (%)	243 (30.6)391 (49.2)	92 (37.9)150 (38.4)	151 (62.1)241 (61.6)	<0.001<0.001

Abbreviations: AFB, acid-fast bacilli; CSF, cerebrospinal fluid; TST, tuberculin skin test.

### Treatment and outcomes

**Figure 3.**
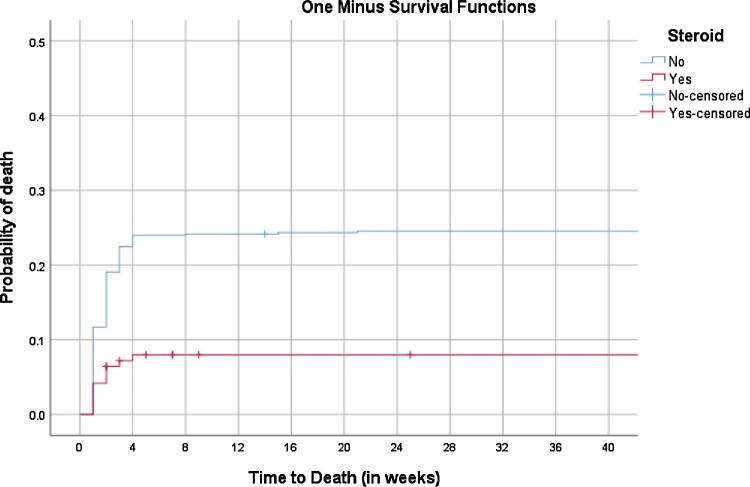
Kaplan–Meier survival analysis for the probability of death in patients who received or did not receive dexamethasone.

Dexamethasone was administered to just 34.0% of children (278/818). Children remained in hospital for 6–34 (median 14) d. At the end of the 12-mo follow-up, 20.2% of TBM patients (160/794) had died, 243/794 (30.6%) were alive with neurological sequelae, while 391/794 (49.2%) were alive without neurological sequelae. The main neurological sequelae were hearing loss (70; 28.9%), epilepsy (61; 25.3%), cerebral palsy (36; 14.7%), hemiplegia (28; 11.6%), paraplegia (22; 9.1%), oculomotor deficit (14; 5.6%) and aphasia (12; 4.8%) (Table 3). Of the 160 deaths, 151 (94.4%) occurred in hospital. The times to death as a function of receiving dexamethasone were rapid for both groups (Figure 3), but the dexamethasone recipients had a threefold increased survival rate, hazard ratio=3.3 (95% CI 2.1 to 5.2, p<0.001).

Upon admission, 156 and 459 children had PCPC scores of 3 and 4, respectively, which improved significantly at 12 mo (Table 4). Moreover, there was a trend of increasing numbers of children with mild disability. However, this increase in mild disability was due to a significant decrease in the number of patients with coma or vegetative state, severe disability and moderate disability.

**Table 4.** Treatment outcomes of 634 surviving TBM patients at different points of time as assessed using the Paediatric Cerebral Performance Category (PCPC) scale

	Follow-up points				
PCPC scale	Admission	1 wk	1 mo	6 mo	12 mo
Normal	4 (0.6)	33 (5.2)	287 (45.2)	405 (63.8)	439 (69.3)
Mild disability	10 (1.6)	43 (6.7)	84 (13.3)	113 (17.9)	114 (17.9)
Moderate disability	125 (19.7)	170 (26.8)	150 (23.6)	58 (9.1)	37 (5.9)
Severe disability	366 (57.8)	297 (46.9)	81 (12.8)	40 (6.3)	27 (4.3)
Coma or vegetative state	129 (20.3)	91 (14.4)	32 (5.1)	18 (2.9)	17 (2.6)

### Risk factors for death

Univariate analysis was performed for 33 risk factors for death. The risk factors that did not show any statistically significant association with death in TBM patients were age, known TB contact, >7 d duration of disease symptoms before admission, low socioeconomic status, malnutrition, cough >2 wk, fever, altered consciousness, clonus, cranial nerve palsy, hypertonia, neck stiffness, raised intracranial pressure (ICP), papilledema, bulging fontanelle, positive AFB smear, abnormal chest radiography, concurrent infections, positive TST, CSF appearance, CSF cells >100/mm^3^, increased CSF protein and decreased CSF glucose. Statistically significant risk factors associated with death in TBM patients were unvaccinated for BCG (p<0.001), not receiving dexamethasone (p<0.001), being male (p<0.001), history of recent weight loss (p<0.001) and MRC stage II and III TBM (p=0.039).

By multivariate logistic regression analysis, the independent risk factors associated with death were (i) unvaccinated for BCG (AOR 3.8, 95% CI 1.8 to 8.1), (ii) not receiving dexamethasone (AOR 2.5, 95% CI 1.5 to 4.2), (iii) being male (AOR 2.3, 95% CI 1.5 to 3.6), (iv) history of recent weight loss (AOR 2.2, 95% CI 1.3 to 3.9) and (v) MRC stage III TBM (AOR 2.0, 95% CI 1.2 to 3.3) (Table 5).

**Table 5.** Univariate and multivariate analyses of the independent risk factors associated with death in children with TBM

		Outcome				
Risk factor	Total (N=794)	Survived (N=634)	Died (N=160)	COR (95% CI)	AOR (95% CI)	p
BCG vaccine^a^VaccinatedUnvaccinated	221 (27.8)573 (72.2)	208 (94.1)426 (74.3)	13 (5.9)147 (25.7)	15.5 (3.1 to 10.0)	3.8 (1.8 to 8.1)	<0.001
Received dexamethasoneYesNo	264 (33.2)530 (66.8)	242 (91.7)392 (74.0)	22 (8.3)138 (26.0)	13.9 (2.4 to 6.2)	2.5 (1.5 to 4.2)	0.001
GenderMaleFemale	314 (39.5)480 (60.5)	212 (67.5)422 (87.9)	102 (32.5)58 (12.1)	3.5 (2.4 to 5.0)1	2.3 (1.5 to 3.6)	<0.001
Recent weight lossPresentAbsent	122 (15.4)672 (84.6)	60 (49.2)574 (85.4)	62 (50.8)98 (14.6)	6.1 (4.0 to 9.2)1	2.2 (1.3 to 3.9)	0.005
TBM stage on admissionStage IStage IIStage III	247 (31.1)435 (54.8)112 (14.1)	205 (83.0)349 (80.2)80 (71.4)	42 (17.0)86 (19.8)32 (28.6)	11.2 (0.8 to 1.8)2.0 (1.2 to 3.3)	1.2 (0.8 to 1.8)2.0 (1.2 to 3.3)	0.3740.013

a BCG vaccination was based on history and documentation of vaccine date.

Abbreviations: AOR, adjusted OR; COR, crude OR; TBM, tuberculous meningitis.

## Discussion

In Afghanistan, a confirmed diagnosis of TBM is very difficult due to limited diagnostic tools and resources. Therefore, TBM diagnosis in our study site was based on the combination of clinical features (e.g. duration of illness) with CSF findings and chest radiography (Marais criteria).^34^ These conditions are common where TBM is most prevalent. There was a long median delay in presentation of approximately 4 wk in our patients, consistent with other studies,^33,41^ and there was favourable response to treatment in many children. Therefore, we believe that most of our children had TBM, cognizant that the differential diagnosis of chronic meningitis is very wide.^41^

Our study is noteworthy for its large size, prospective nature and high proportion of children aged <5 y who are underrepresented in some studies. We did not set out to examine specifically the effect of dexamethasone and dexamethasone recipients had several baseline characteristics that were significantly different vs the non-dexamethasone group. Nevertheless, dexamethasone significantly reduced mortality by approximately 70% in those with MRC grade III.

Randomised trials confirming the mortality reduction of dexamethasone in TBM have been reported in Vietnamese adolescents and adults,^42^ in ‘young’ South African children (ages not specified)^32^ and Egyptian patients across the age spectrum^31^; a later retrospective study of 300 Egyptian children aged <5 y also found reduced mortality in steroid recipients.^33^ These results are consistent with several paediatric studies from India^30,43,44^ and Vietnam,^45^ but not with one Thai study.^46^ A Cochrane review of nine TBM studies found an overall mortality benefit of steroids of 25% in HIV-1–negative adults and children, but their effect on reducing disabling neurological deficits remains open and requires more research.^47^

Afghanistan's health system is facing serious challenges, mostly due to insecurity. This has negatively affected the treatment outcome of all forms of TB in Afghanistan, especially in rural areas, where the security situation is worse. In our study, the treatment success rate was 79.8%. A retrospective cohort study (2011–2014) on all forms of TB was conducted in a war-affected region of Khyber Pakhtunkhwa in the northwest of Pakistan. This study revealed that among 181 extra-pulmonary TB (including TBM) patients, 80.1% had successful treatment outcomes.^48^ This treatment success rate is nearly the same as observed in our study.

Our overall mortality rate was high, approximately 20%, which is higher than the mortality rate reported in childhood TMB from China (4.0%),^2^ Thailand (6.4%),^49^ Romania (8.0%),^50^ South Africa (13.0%)^51^ and Vietnam (15.7%),^3^ but lower than in India (29.0%)^4^ and Ethiopia (48.1%).^52^

In our study, worryingly, most deaths occurred soon after admission and the identified, independent risk factors associated with death were male gender, recent weight loss, no BCG vaccination and no dexamethasone given in hospital. TBM mortality risk factors vary across settings but several key factors have been consistently identified, namely, more severe disease at presentation,^53,54^ which, in turn, is related to delayed presentation and diagnosis,^5^^2^,^5^^5–^^57^ young age,^53^ HIV positivity,^58–60^ the lack of BCG vaccination,^61–63^ low socioeconomic status^9^ and the presence of raised ICP.^64^ In Afghanistan, additional factors for mortality can probably be linked to Afghanistan's fragile healthcare system, national insecurity, the unavailability of advanced life support for seriously ill patients, misdiagnosis and poor referral linkage between primary care and tertiary centres.^65^

Receiving BCG vaccination at birth has a reported efficacy of approximately 73%^66^ and it has been estimated that had BCG vaccinations been given to 100.5 million children born in 2002, some 30 000 TBM cases would have been prevented (one case/3500 inoculations).^66^ However, one Indian study reported a reduced benefit in children aged >5 y with severe malnutrition and a positive household contact.^67^ Another effective strategy is post-exposure prophylaxis in young (aged <5 y) children,^68^ but none of our children received this, illustrating again the significant gap between policy and practice in a developing country.^4,69^

Our study had several limitations. It was conducted at a tertiary referral hospital and this may have introduced a mortality bias, because the severity spectrum of TBM we observed may not be seen in smaller district hospitals. The nature of the study is biased by having the diagnosis of TBM already assigned to the cohort. Although Mirwais regional hospital is a referral hospital, its diagnostic capacity is limited and all TBM diagnoses were made clinically with supporting evidence from CSF findings and chest radiograph; we do not have facilities for TB culture or neuroimaging. We did not screen for HIV status because the prevalence of HIV is very low in Afghanistan and HIV tests are not performed routinely for inpatients.

Diagnostic tests and treatment in Mirwais regional hospital are free of charge and attract patients from all the districts of Kandahar province, as well as the neighbouring provinces of Helmand, Uruzgan, Zabul, Farah and Nimruz. With such a wide catchment area, challenges with transport may have introduced delays in patients’ presentation.

## Conclusions

TBM continues to cause high morbidity and mortality in Afghan children and, in our setting, death occurred rapidly. Administering BCG vaccination and giving dexamethasone to all TBM patients are two simple and inexpensive interventions that could have a profound impact on TBM-related mortality. Our data support the use of dexamethasone for 4 wk, but future research should determine the optimal dose of dexamethasone in vulnerable children aged <5 y.

## Data Availability

Data is available on request to the email drbilal77@yahoo.com.
